# COVID-19 Experience: Taking the Right Steps at the Right Time to Prevent Avoidable Morbidity and Mortality in Nigeria and Other Nations of the World

**DOI:** 10.2147/IJGM.S261256

**Published:** 2020-08-04

**Authors:** Obinna O Oleribe, Princess Osita-Oleribe, Babatunde L Salako, Temitope A Ishola, Michael Fertleman, Simon D Taylor-Robinson

**Affiliations:** 1 Excellence and Friends Management Care Centre, Abuja, Nigeria; 2 Centre for Family Health Initiative, Abuja, Nigeria; 3 Nigeria Institute for Medical Research, Lagos, Nigeria; 4 Department of Biochemistry, Covenant University, Ota, Nigeria; 5 Cutrale Perioperative & Ageing Group, Department of Bioengineering, Imperial College London, London, UK; 6 Department of Surgery and Cancer, Imperial College London, London, UK

**Keywords:** Nigeria, COVID-19, pandemic, prevention

## Abstract

The 2020 Coronavirus pandemic has caused countless governmental and societal challenges around the world. Nigeria, Africa’s most populous nation, has been exposed in recent years to a series of epidemics including Ebola and Lassa fever. In this paper, we document our perception of the national response to COVID-19 in Nigeria. The response to the pandemic is with a healthcare system that has changed as a result of previous infectious disease outbreaks but in the context of scarce resources typical of many low-middle income countries. We make recommendations regarding what measures should be in place for future epidemics.

## Background

On 11 March 2020, the World Health Organization (WHO) declared a pandemic caused by Coronavirus disease (COVID-19), an infectious disease caused by severe acute respiratory syndrome coronavirus 2 (SARS-CoV-2).^1^ Since first being detected Wuhan, China in December 2019, the disease has rapidly escalated to over 210 countries, leading WHO to declare the outbreak a “Public Health Emergency of International Concern” on 30 January 2020 and eventually a pandemic within 6 weeks.^2^–^5^ Globally, as of April 30, 2020, there have been 3,090,445 confirmed cases of COVID-19 and 217,769 related deaths giving a case mortality rate of 7.0%.^6^ The pandemic took less than 100 days to exceed one million confirmed cases by April 2, 2020.^7^

The disease was first diagnosed in Nigeria on 27 February 2020. As of April 30, 2020, diagnostic tests had been conducted on 15,759 samples with 1932 confirmed cases. Three hundred and nineteen patients had made a full recovery and 35 had died, in test positivity, recovery, and case fatality rates of 12.3%, 16.3% and 1.8%, respectively, throughout the country.^8^

Globally governments have attempted to curtail the pandemic and curb its spread; with national lockdown, social distancing, regular handwashing, stay at home and self-isolation policies.^9^ These policies were captured in the WHO 2019 Novel Coronavirus (2019-nCoV): Strategic Preparedness and Response Plan, an objective of which is “to stop further transmission of 2019-nCoV within China and to other countries, and to mitigate the impact of the outbreak in affected countries.”^10^

As the world comes to terms with this pandemic we begin to wonder if there anything that could be done better to minimize morbidity and mortality associated with the next pandemic. Currently with more than three million cases and 200,000 deaths, there is a need to review and identify what strategies and activities could be started, continued or stopped, or continued more effectively and efficiently to minimize the impact of the next pandemic.

This paper will discuss the strategies that were applied in Nigeria and those that should be applied in the future.

## Strategies Adopted in Nigeria

In Nigeria, the Federal Ministry of Health through the Nigeria Centre for Disease Control (NCDC) took steps to control COVID-19 in line with its mandate. The National Emergency Outbreak Committee (EOC) was established and developed the Incident Action Plan (IAP) on March 27, 2020 with eight pillars – Epidemiology and Surveillance, Laboratory, Point of Entry (POE), Infection Prevention and Control, Case Management, Risk Communication, Logistics, and Coordination.

Within 2 months, NCDC developed several guidelines and protocols, including NCDC Self-Isolation Guideline for COVID-19, Case Definition for Coronavirus, PPE Recommendations During Health Care Delivery, National COVID-19 Case Management Guide NCDC, Guide for Businesses for COVID-19, Social Distancing Guide for COVID-19, Infection Prevention and Control Guideline for COVID-19, Rational Use of Personal Protective Equipment in the Care of COVID-19 Cases, Management of Dead Bodies, Advisory on Use of Face Masks, and COVID-19 Testing Strategy.^11^ Meetings were held daily by the EOC and meetings were held in individual states to curtail the outbreak. Hospitals were assessed and activated as isolation and treatment centers, laboratories were equipped to test for the virus using PCR technology and staff were trained to manage cases, test patients, track contacts, and document findings.

## Reflections and Recommendations

Despite all these measures, at present, the number of cases continues to rise. This has led us to review our current practice to identify what can be put in place to prevent similar epidemics in the coming years. In addition, we present some immediate strategies that could reduce COVID-19 infection.

### Quick and Immediate Assessment

Early in any outbreak, there is need to conduct a quick and immediate assessment of resources and current activities. This assessment should appraise available resources, and all relevant public health activities and outputs. Sample questions that may be asked include what resources are available; what is needed to manage the outbreak; what are the gaps; what activities have been executed including what did and did not work; what new activities can be introduced; what can we stop; what can we modify; and what lessons can we learn from elsewhere? These questions will also analyze the strength, weaknesses, opportunities and threats of the control system and processes. This will provide background information for the next phase of programing.

### Establishment of Regional or State Incident Management Teams (IMTs)

Beyond a National Incident Management Team (NIMT), there is a need to concurrently establish Regional or State Incident Management Teams (R/SIMT). Each team will include the Regional/State Epidemiologist, highly experienced Laboratory Manager, Communication Officer, Chief Medical Officer of a reference facility or tertiary hospital in the region/state, and a Finance Officer/manager. At the national level, a National IMT will comprise a Director, Presidential Task Force; Director General, National Centre for Disease Control; Director, Emergency and Preparedness; Project Manager/Consultant; Communication Officer and Research Lead. These incident managers must be quickly constituted, mobilized and operationalized proactively to ensure immediate roll out of protocols, guidelines and standard operating procedures like those developed in Nigeria by the Nigeria Centre for Disease Control (NCDC).^11^

### Capacity Development of IMTs

Capacity development of all members of the IMT is critical to ensure the quality of services provided, safety of the frontline workers and health of patients. All IMTs should be trained on developed guidelines, protocols, standards of practice, and algorithms. All training should be virtual to minimize physical contact and traveling. This training should be facilitated by highly qualified persons from international organizations where available and relevant university departments in the country, region or state. In-person training should be reduced to the barest minimum.^12^

### Establishment of Testing and Diagnosis

To control an outbreak, screening and testing must be available. Where the resources are available, Polymerase Chain Reaction (PCR) empowered Laboratories should be established in each region/state. These can be established in partnership with the governments of the regions/states and corporate organizations. Regions/States with the capacity should establish one laboratory per county/Senatorial District. Eventually, the development of antigen-antibody testing can minimize the use of (PCR) techniques which are usually expensive and require a high level of technical skill. As quickly as possible, states should move from facility-based PCR to community-based antigen-antibody testing using rapid test kits. Each outbreak should be an opportunity to develop the capacity of local partners to create new testing centers and build the capacity of healthcare workers to screen and test.

To support the testing and diagnosis facilities, Sample Collection Centers (SCC) should be established in all communities across the nation. Simple test strips should be introduced as quickly as possible for screening purposes, and positive samples referred for confirmation at the nearest specialist center.

### Fund Generation and Drive

Funds are needed to manage outbreaks particularly to protect the vulnerable communities.^13^ Budgetary allocations from the national government can jumpstart the process. However, both the IMTs and control organizations should be fast in seeking and obtaining funds from non-government sources. Donors can reprogram funds towards the management of the outbreak. Regions, states, corporate organizations and individuals can fund the establishment of laboratories, procurement of test kits, training of healthcare workers, engagement of volunteers and even establishment of isolation and quarantine centers.

### Establishment of Diagnosis, Isolation and Treatment (DIT) Centers

Beyond screening and stand-alone testing sites, outbreaks like COVID-19 should have integrated management systems. This will involve the establishment of integrated Diagnosis, Isolation and Treatment (DIT) centers in all Regions/States where none exist. The goal would be to have a DIT center within a 10-km radius. This can be started with one per region/state. Self-isolation should be discouraged, and exposed people should be asked to be isolated in government-designated DIT locations. New DITs could be located in community halls, open spaces, stadia, hotels, etc. This strategy is designed to take the outbreak (like COVID-19) out of the hospitals. Taking the outbreak out of the hospitals will allow the secondary and tertiary hospitals to manage the conventional illnesses and diseases common in each locality. Each DIT can have from 50 to 200 beds to accommodate isolation cases. DIT centers will decentralize testing, isolate and manage cases, as well as relieve the burden on hospital facilities across the nations of the world.

### Human Capacity Mobilization

To overcome a global outbreak, healthcare workers should be mobilized and utilized strategically to achieve the agenda of expanding testing, preventing infection, and forming effective partnerships in the community. This requires mobilization of volunteer healthcare workers and other support-staff from relevant organizations. Doctors, nurses, pharmacies, laboratory scientists, community health workers, and support staff should be mobilized to volunteer as frontline workers to manage the DIT centers in each location. More volunteers will be placed where they are needed most. Volunteers should be managed and supervised by the relevant IMTs and trained virtually. Trained volunteers and healthcare workers should be empowered and supported to train others, scale up and scale out services. They will also help to screen at community level, track contacts of confirmed cases and participate in facility readiness assessment. Finally, they should be engaged in community education and information management.

### Information Management

Information management is critical to outbreak management and control. Using relevant technological applications and platforms, up to date information on an outbreak locally and nationally can be monitored. Also, through surveys such as the BroadReach Vantage^14^, an assessment can be made of the impact of the intervention, community knowledge base, and attitude and practice of people.

### Research and Publications

Confirmed cases can be used to conduct trials to understand causality analysis, what is effective, and experiment with different antiviral/antibiotics/antiparasitic combinations to identify what works best among the population. It should be possible to collect data about complications, length of stay in hospital, antigen and antibody conversion rates and factors that influence them; and investigate factors associated with various degrees of infection and manifestations. Publications will promote global information sharing and allow nations just beginning to experience the outbreak to know what works to help curb the spread of the virus.

### Monitoring and Evaluation

The various strategies cannot stand alone but can be executed concurrently based on the identified and agreed timelines. There should be daily virtual meetings by the IMT to assess progress and make mid-course corrections; weekly virtual meetings to generate new ideas and review weeklong activities while finalizing the next week project; weekly publications of activities, finances, and lessons learned; and weekly M&E questionnaire completion and analysis.

Active use of everyday data gathered during the process of the outbreak investigation, the clinical presentation and epidemiology of the disease can be readily characterized including the use of data science, projections/modelling and data visualization.

## Conclusion

The Nigerian government responded to the COVID-19 pandemic by various measures affecting the population and the creation of necessary national teams to provide structure and leadership. These teams interfaced with the country to discuss the plan for treatment and control of the pandemic in Nigeria. In the recovery from COVID-19 medical authorities need to integrate their responses in a coordinated and streamlined way to prevent silo or vertical reactions to crises. Lateral and coordinated thinking is required. The lessons learnt from the COVID-19 pandemic need to be implemented in policy documents to ensure readiness to prevent future, similar world crises. We need to build the relevant structures and partnerships to ensure we fight locally and win globally.
